# Predictors of postendoscopic retrograde cholangiopancreatography pancreatitis, analysis of more than half a million procedures performed nationwide over the last 15 years

**DOI:** 10.1002/jgh3.12341

**Published:** 2020-04-17

**Authors:** Ali Abbas, Sajiv Sethi, Gitanjali Vidyarthi, Pushpak Taunk

**Affiliations:** ^1^ Section of Gastroenterology USF Health Morsani College of Medicine Tampa Florida USA; ^2^ Section of Gastroenterology James A Haley VA Hospital Tampa Florida USA; ^3^ Division of Digestive Diseases and Nutrition University of South Florida Morsani College of Medicine Tampa Florida USA

**Keywords:** complications, endoscopic retrograde cholangiopancreatography, pancreatitis, post‐endoscopic retrograde cholangiopancreatography pancreatitis, nationwide, epidemiology, health care quality indicators

## Abstract

**Background and Aim:**

Post‐ERCP pancreatitis (PEP) is the most common complication following endoscopic retrograde cholangiopancreatography (ERCP). It is still controversial whether the presence of a trainee would increase the risk of PEP. Additionally, the effects of demographic factors and comorbidities on the risk and severity of PEP are not fully understood. Our aim was to evaluate these factors using national database.

**Methods:**

Nationwide Inpatient Sample 2000–2014 was used to identify adult patients admitted with biliary obstruction without acute pancreatitis and had an inpatient ERCP. PEP was defined as having a subsequent diagnosis of acute pancreatitis. The presence of major organs failure marked moderate–severe PEP. Demographic information, hospital characteristics, and ERCP intervention types were collected.

**Results:**

We included 654 394 patients. Overall PEP rate was 5.4%. The PEP rate was lower in teaching (4.8%) compared with nonteaching (6.2%, *P* < 0.001) hospitals. The highest PEP rate was observed among patients undergoing Sphincter of Oddi Manometry (15.1%, odds ratio [OR] = 2.5, *P* < 0.001) as compared to diagnostic cholangiography (4.4%). Asians and Hispanics had higher rate of PEP (10% and 7.9%, respectively) compared with Caucasians and African Americans (4.9% and 5%, respectively, *P* < 0.001). Multivariate analysis showed that after controlling for the ERCP intervention types, Asians and Hispanics continued to have higher odds of PEP (OR = 1.3, *P* < 0.001). Seventeen percent of patients were classified as moderate–severe PEP. Older patients (OR = 3.2, *P* < 0.001), males (OR = 1.4, *P* < 0.001), and high comorbidities (1.3, *P* < 0.001) were major predictors of moderate–severe PEP.

**Conclusion:**

No evidence of higher PEP rates in teaching hospitals. Asians and Hispanics had higher PEP rates. Although ERCP intervention type is the major PEP predictor, its severity is dependent on patient characteristics.

## Graphical abstract

Post‐ERCP Pancreatitis (PEP) is the most common complication following ERCP. Additionally, the effects of demographic factors and comorbidities on the risk and severity of PEP are not fully understood. Nationwide Inpatient Sample 2000–2014 was used to identify adult patients admitted with biliary obstruction without acute pancreatitis and had an inpatient ERCP. We included 654 394 patients and found no evidence of higher PEP rates in teaching hospitals. Asians and Hispanics had higher PEP rates. We note that ERCP intervention type is the major PEP predictor, and its severity was dependent upon patient characteristics.

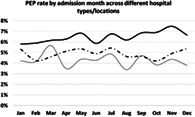

## Background

Post‐ERCP pancreatitis (PEP) is the most common complication following endoscopic retrograde cholangiopancreatography (ERCP) procedure.^1^, ^2^ The reported incidence of PEP ranges from 2 to 10% (2–4% in low‐risk populations and 8–40% in high‐risk groups).^3^, ^4^, ^5^ A recently published meta‐analysis reported an overall incidence rate of 9.7% and mortality of 0.7%.^6^ PEP is mostly mild to moderate, but in some cases, it could lead to prolonged ICU admission and death.^6^ It is estimated that PEP costs $150–$200 million yearly in the United States.^6^, ^7^

There are multiple factors that could potentially increase the risk of PEP, such as patients' related factors (younger age, female gender, sphincter of Oddi dysfunction, prior PEP, normal serum bilirubin, and recurrent pancreatitis).^3^, ^4^, ^8^, ^9^ Other reported factors include provider related factors (low volume center and involvement of trainee) and procedure‐related factors (difficult cannulation, pancreatic duct cannulation or contrast injection, precut sphincterotomy, and ampullectomy).^1^, ^3^, ^4^, ^8^, ^9^, ^10^, ^11^, ^12^ These factors could play an additive or synergistic role in the risk of PEP and thus multivariate analysis is essential to identify their respective independent effect.^6^

While some risk factors are well established, others remain controversial, mainly the endoscopist experience, hospital volume, and trainee involvement.^1^, ^4^, ^10^ Additionally, a recent meta‐analysis indicated global variation in the rate of PEP across trials from North America, Europe, and Asia, suggesting the presence of additional racial and demographic risk factors that are yet to be investigated.^6^ Furthermore, among patients with PEP, the predictors of severe PEP are still unknown.

Our aim is to investigate the predictors of PEP, specifically moderate–severe PEP, using a national database to better understand the racial and geographic variations after accounting for the classic procedure‐related factors. We also aimed to compare PEP across teaching and nonteaching centers to explore the potential effect of trainee involvement early in the academic year.

## Methods

### *Data source*

We obtained data from the Nationwide Inpatient Sample (NIS) from 2000 to 2014. The NIS is a component of the Healthcare Cost and Utilization Project (HCUP),^13^ sponsored by the Agency for Healthcare and Quality. This database represents the largest all‐payers inpatient database in the United States. The NIS does not contain any patient identifiers and therefore, does not require institutional review board approval before use; however, it requires all users to complete the Healthcare Cost and Utilization Project data use agreement training before performing any scientific analysis. When the number of patients reported in a category is less than 10, the exact proportion is not reportable to maintain patient confidentiality; however, the reported p value is calculated with the exact number of observations in the analysis.

### *Inclusion criteria*

We included all adult patients who were admitted nationwide between 2000 and 2014 with biliary obstruction without pancreatitis and had an inpatient ERCP. These patients met all the following criteria: (i) admitted with primary or secondary ICD9 diagnosis code of biliary duct obstruction, (ii) had no primary or secondary diagnosis of acute pancreatitis, and (iii) had ERCP done during that admission.

Patients with codes of chronic pancreatitis or pancreatic pseudocyst were excluded from the analysis, as a reliable diagnosis of PEP cannot be made. Additionally, given that both cholangitis and moderate–severe pancreatitis are characterized by sepsis and organ failure independently, it would be difficult to determine if those clinical outcomes were due to cholangitis or pancreatitis. Thus, patients with cholangitis were also excluded from the study.

### *Outcomes and study design*

Our primary outcomes were the occurrence of PEP (all severity level for the first analysis), predictors of moderate–severe PEP (for the second analysis), and in‐hospital mortality following inpatient ERCP for biliary obstruction. PEP was defined as having a subsequent ICD9 code of acute pancreatitis during the same admission (not primary or secondary diagnoses for the encounter). We adopted this definition from prior published studies that investigated PEP using administrative database.^14^, ^15^

Among patients who were identified as PEP, moderate–severe PEP was identified by the presence of ICD9 diagnosis codes of major organ failure or complications (acute renal failure, respiratory failure, sepsis, or shock) during hospitalization. This definition was adopted from Atlanta Classification that defined moderate/severe/critical pancreatitis by the presence of transient or persistent organ failure and/or sepsis from infected peripancreatic collection.^16^ In‐hospital mortality is also provided by the NIS database.

We conducted a cross‐sectional retrospective database analysis. Incidence rates of PEP, moderate and severe PEP, and in‐hospital mortality following inpatient ERCP for biliary obstruction were calculated and compared across different potential predictors. Monthly rates of PEP were calculated and then stratified by hospital location and teaching status (NIS classifies hospitals to rural, urban‐nonteaching, and urban‐teaching hospitals) to clarify if ERCPs done early in the academic year at teaching hospitals were associated with higher rates of PEP.

### *Other identified predictors*

ERCP intervention types were identified using the specific ICD9 procedure codes. Then ERCP interventions were classified into four major groups: (i) biliary intervention (sphincterotomy, stone extraction, dilation, stent placement, or Sphincter of Oddi manometry), (ii) pancreatic intervention (pancreatic duct stone extraction, dilation, or stent placement), (iii) patients were classified to having both interventions if they had biliary and pancreatic interventions, and (iv) those who had no interventions were classified as diagnostic ERCP (included patient who had cholangiography, pancreatography, and pancreatic duct wire cannulation without pancreatography). The analysis was performed first by including the individual interventions to estimate the odds ratio of PEP for each specific intervention, and then the analysis was repeated by including ERCP intervention groups to account for multiple procedures within the same patient.

Other variables including demographics (age, sex, race), geographic area (west, northeast, midwest, and south), insurance (private, Medicare, Medicaid), year and month of admission, day of admission (weekend *vs* not weekend), and hospital characteristics (per NIS database, urban hospital located in metropolitan statistical areas, and teaching designation from the presence of AMA approved residency program), were identified. Comorbidities were identified using ICD9 codes and summarized using Charlson Comorbidity Index (CCI) score.^17^ Patients were classified to two groups 0–1 and >1 based on Charlson comorbidity scores. ICD9 codes used in this analysis presented in the Supporting information.

### *Statistical analysis*

Univariate analysis was performed to identify potential predictors of our outcomes (PEP and moderate–severe PEP) using chi square, Fisher exact, and Student's *t*‐test when appropriate. Age was classified into 18–39, 40–64, and >64 in the multivariate models. Using predictors that achieved statistical significance (*P* < 0.05) in the univariate analysis, multivariable logistic regression analysis with backward stepwise selection of the predictors keeping statistically significant and clinically relevant predictors was performed to assess for independent predictors of our outcomes. Data were analyzed by using the SAS software, Version 9.4 for Windows (SAS Institute Inc., Cary, NC, USA), licensed to University of South Florida. We took into account the samplings weights provided with the NIS database when performing the analysis.

## Results

A total of 654 394 patients who were admitted nationwide between 2000 and 2014 with biliary obstruction and underwent inpatient ERCP were included in the analysis. The median age was 59 years (interquartile range of 39–76) with females representing 66% of the cases. Overall, the PEP rate was 5.4% (35 395 patients), Table 1. The overall in‐hospital mortality rate for the included population undergoing ERCP was 0.7%. Whereas patients with PEP had a higher mortality rate of 1.2% (*P* < 0.001), this varied by severity of PEP (mild PEP was 0.3% compared to 6.2% for moderate–severe PEP, *P* < 0.001).

**Table 1:** Descriptive parameters of the included population

		Total	%
Age	18–39 years old	166 912	25.5
	40–64 years old	208 362	31.8
	≥65 years old	279 119	42.7
Race	White	393 028	60.1
	Black	46 614	7.1
	Hispanic	106 265	16.2
	Asian	19 096	2.9
	Others/unknown	89 367	13.7
Gender	Female	431 669	66.0
	Male	222 724	34.0
Insurance	Private	223 275	34.1
	Medicare	274 634	42.0
	Medicaid	83 231	12.7
	Others/self‐pay	73 253	11.2
Charlson Comorbidity Index	CCI 0–1	503 783	77.0
	CCI >1	150 610	23.0
PEP	No PEP	618 999	94.6
	Mild PEP	29 502	4.5
	Moderate/Severe PEP	5893	0.9
ERCP intervention group	No intervention	70 275	10.7
	Biliary intervention	560 239	85.6
	Pancreatic intervention	3189	0.5
	Both biliary and pancreatic	20 691	3.2
Academic year	July–December	311 758	50.4
	January–June	306 581	49.6
Admission day	Weekday	517 081	79.0
	Weekend	137 313	21.0
Location/teaching status of hospital	Rural	44 575	7
	Urban‐nonteaching	291 060	45
	Urban‐teaching	316 551	49
Region of hospital	Northeast	157 852	24.1
	Midwest	94 843	14.5
	South	250 353	38.3
	West	151 346	23.1

ERCP, endoscopic retrograde cholangiopancreatography; PEP, post‐ERCP pancreatitis.

### *Rate of PEP by teaching status of the hospitals*

**Figure 1:**
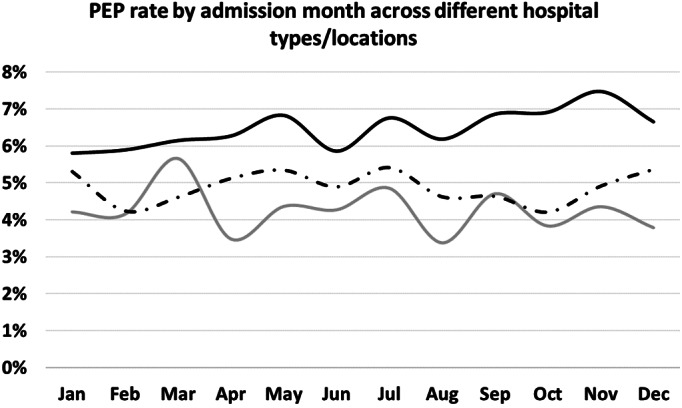
Post endoscopic retrograde cholangiopancreatography (ERCP) pancreatitis rate by admission month across different hospital types/locations, 338 × 190 mm (96 × 96 DPI). 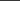, rural; 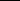, urban nonteaching; 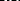, urban teaching.

**Figure 2:**
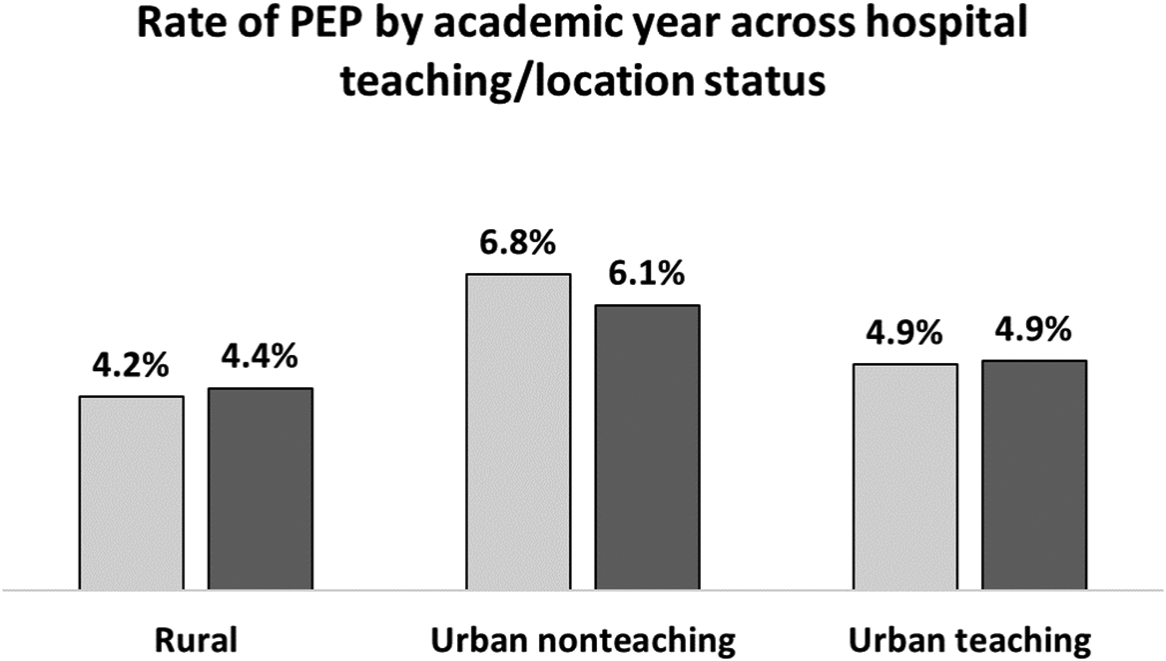
Rate of post‐ERCP pancreatitis (PEP) after endoscopic retrograde cholangiopancreatography (ERCP) with intervention by academic year across hospital teaching/location status, 338 × 190 mm (96 × 96 DPI).

Among the included patients, 49% were admitted to urban‐teaching hospitals, 45% to urban‐nonteaching, and 7% to rural hospitals. PEP rates varied significantly across hospital types, with rural having the lowest rate of PEP at 4.2%, followed by urban‐teaching at 4.8% and the highest rate in urban‐nonteaching at 6.2% (*P* < 0.001). No statistically significant difference was observed in the monthly rate of PEP in teaching hospitals (Fig. 1). ERCP with intervention was performed in 89% of the cases. Analysis of PEP rates of interventional ERCP also showed no significant difference for the period of July–December as compared to January–June for rural and urban‐teaching hospitals. However, among patients who were admitted to urban‐nonteaching hospitals, the PEP rate was slightly higher during July–December period (7%) compared to January–June (6.3%, *P* < 0.001) (Fig. 2). Thus, the month during which ERCP was performed was excluded from subsequent analysis predicting PEP.

### *Predictors of PEP*

**Figure 3:**
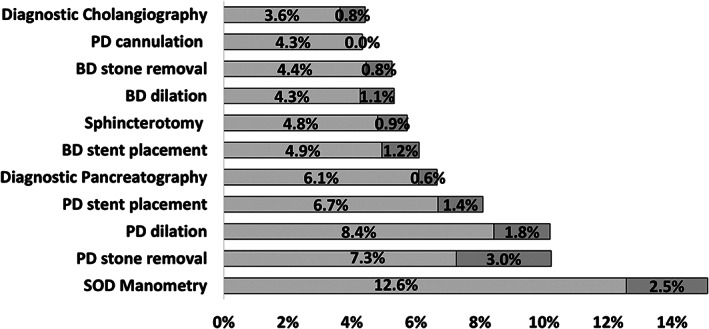
Rate of post‐ERCP pancreatitis (PEP) and moderate–severe PEP by endoscopic retrograde cholangiopancreatography (ERCP) intervention type. BD, bile duct; ERC, endoscopic retrograde cholangiography; ERP, endoscopic retrograde pancreaticography; PD, pancreatic duct; SOD, Sphincter of Oddi.

PEP rates varied significantly across the type of ERCP intervention with Sphincter of Oddi Dysfunction (SOD) manometry having the highest rate at 15.1%, whereas diagnostic cholangiography and pancreatic duct wire cannulation had the lowest 4.4% and 4.3% respectively (*P* < 0.001), Figure 3. On multivariate analysis, patients who had SOD manometry had the highest risk of having PEP (odds ratio [OR] = 2.58, *P* < 0.001), whereas patients who had diagnostic cholangiography or pancreatic duct wire cannulation were not at higher risk of PEP (*P* = 0.9). PEP rate of those who underwent diagnostic cholangiography was 4.4% (OR 1, *P* = 0.9), representing the baseline risk group for PEP. Interestingly, those who had bile duct stone removal or bile duct stricture dilation were at lower risk of PEP (Table 2). When analyzing by intervention group, patients who had both biliary and pancreatic interventions had a higher rate of PEP (8.3%, OR = 2, *P* < 0.001) compared to diagnostic ERCPs (4.5%) (Table 2).

**Table 2:** Multivariate analysis predicting PEP based on demographics and ERCP intervention

		Total #	PEP #	%	OR	LCI	UCI	*P*
Race	Caucasians	393 028	19 126	4.9				
	African American	46 614	2318	5.0	1.21	1.16	1.27	<0.001
	Hispanic	106 265	8360	7.9	1.26	1.22	1.30	<0.001
	Asian	19 096	1910	10.0	1.25	1.19	1.32	<0.001
	Others/unknown	89 367	3682	4.1	0.88	0.85	0.92	<0.001
ERCP intervention group	No intervention	70 275	3134	4.5				
	Biliary	560 239	30 312	5.4	1.19	1.14	1.24	<0.001
	Pancreatic	3189	237	7.4	1.98	1.72	2.28	<0.001
	Both	20 691	1712	8.3	2.01	1.89	2.15	<0.001
ERCP detailed intervention (entered to the analysis after removing ERCP groups intervention variable)	Diagnostic cholangiography	68 518	3022	4.4	1.00	0.95	1.06	0.976
	PD cannulation	116	LN	LN	1.05	0.42	2.60	0.914
	BD stone removal	372 545	19 570	5.3	0.86	0.84	0.89	<0.001
	BD dilation	38 179	2030	5.3	0.93	0.89	0.98	0.003
	Sphincterotomy	487 061	27 885	5.7	1.31	1.27	1.36	<0.001
	BD stent placement	172 871	10 549	6.1	1.16	1.13	1.19	<0.001
	Diagnostic pancreatography	1732	115	6.7	1.64	1.35	2.00	<0.001
	PD stent placement	22 736	1839	8.1	1.60	1.52	1.68	<0.001
	PD dilation	830	85	10.2	1.85	1.47	2.33	<0.001
	PD stone removal	840	86	10.2	1.67	1.33	2.10	<0.001
	SOD Manometry	197	30	15.1	2.58	1.72	3.87	<0.001

# number of patients. ERCP, endoscopic retrograde cholangiopancreatography; PEP, post ERCP pancreatitis; OR, odds ratio; LCI, UCI, lower and upper limits of the 95% confidence interval of the OR. LN, low number (below 10), omitted per NIS database guidelines. The analysis is adjusted for age, gender, insurance, weekend admission, hospital teaching and urban status, hospital region, Charlson Comorbidity Index (not shown in the table, refer to the Supporting information for full table).

Asians and Hispanics had higher rate of PEP (10% and 7.9% respectively) compared to Caucasians and African Americans (4.9% and 5% respectively, *P* < 0.001). Patients from western states had the highest rate of PEP (11.7%) compared with patients in southern states (3.2%, *P* < 0.001). Multivariate analysis showed that after controlling for the ERCP intervention, Asians and Hispanics continued to have higher odds of PEP as compared to Caucasians (around 1.3, *P* < 0.001). Other factors that were independently associated with higher rates of PEP included west geographic region (OR 3.4, *P* < 0.001), Table 2. Full table of the analysis is presented in the Supporting information.

### *Predictor of moderate–severe PEP*

**Figure 4:**
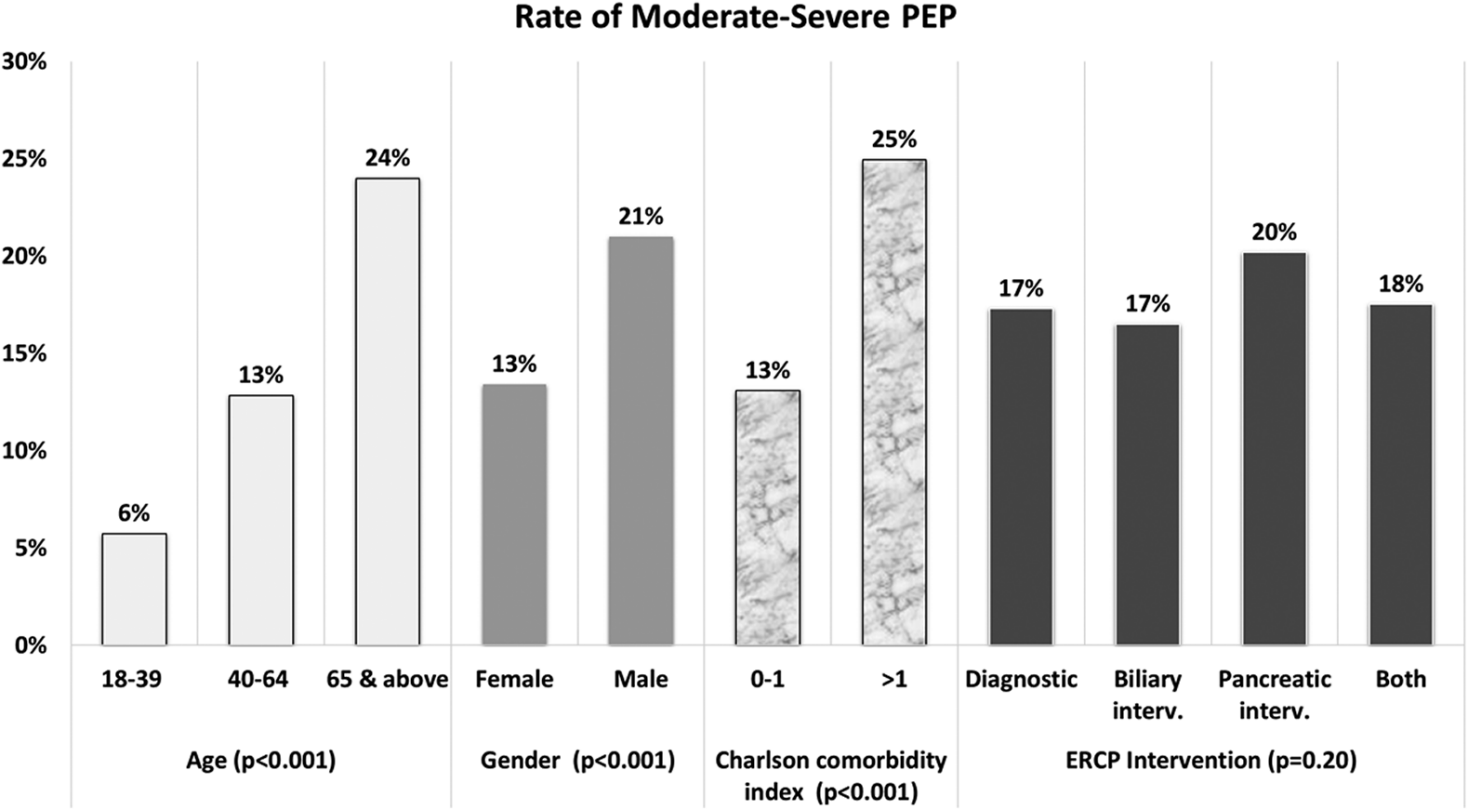
Rate of moderate–severe post‐ERCP pancreatitis (PEP) across different demographic and procedure‐related risk factors, 338 × 190 mm (96 × 96 DPI).

A total of 35 395 patients with PEP were included in the analysis. Among them, 17% were classified as moderate–severe PEP (0.9% of all ERCPs). Older age, male gender, and patients with high comorbidities had almost double the rate of moderate–severe PEP compared with other patient groups (*P* < 0.001). Interestingly, the type of ERCP intervention performed was not a predictor of moderate–severe PEP, with diagnostic ERCP (17%) as compared to those who had both biliary and pancreatic interventions (18%, *P* = 0.208), Figure 4. ERCP intervention was thus excluded from multivariate analysis. Multivariate analysis showed that older age (≥ 65 years old, OR 3.2, *P* < 0.001), male gender (OR 1.4, *P* < 0.001), and higher comorbidities (CCI >1, OR 1.3, *P* < 0.001) were the main independent predictors of moderate–severe PEP (Table 3).

**Table 3:** Multivariate analysis predicting moderate to severe PEP

		Total #	Mod‐Sev PEP #	%	OR	LCI	UCI	*P*
Age	18–39 years old	8463	510	6				
	40–64 years old	11 531	1530	13	1.9	1.7	2.1	<0.001
	≥65 years old	15 401	3852	25	3.2	2.8	3.7	<0.001
Race	White	19 126	3775	20				
	Black	2318	460	20	1.1	1.0	1.2	0.137
	Hispanic	8360	819	10	0.8	0.7	0.9	<0.001
	Asian	1910	271	14	0.9	0.8	1.0	0.081
	Others/unknown	3682	567	15	0.8	0.7	0.9	<0.001
Gender	Female	23 784	3348	14				
	Male	11 611	2545	22	1.4	1.3	1.4	<0.001
Insurance	Private	11 381	1327	12				
	Medicare	15 009	3737	25	1.3	1.1	1.4	<0.001
	Medicaid	5719	502	9	1.0	0.9	1.1	0.782
	Others/self‐pay	3286	327	10	1.0	0.8	1.1	0.503
Charlson Comorbidity Index (CCI)	CCI 0–1	26 724	3668	14				
	CCI >1	8671	2225	26	1.3	1.2	1.4	<0.001
Region of hospital	Northeast	5657	1187	21				
	Midwest	3837	715	19	0.8	0.8	0.9	0.002
	South	8136	1978	24	1.2	1.1	1.3	<0.001
	West	17 765	2013	11	0.6	0.5	0.6	<0.001

# number of patients. Mod‐Sev, moderate to severe; PEP, post ERCP pancreatitis; OR, odds ratio; LCI, UCI, lower and upper limits of the 95% confidence interval of the OR.

## Discussion

In the nationwide study that analyzed more than half a million inpatient ERCPs performed for biliary obstruction, there was no evidence of trainee involvement as a risk factor for higher rate of PEP. Additionally, there were racial and geographic variations in the PEP rate even after controlling for the classical procedure‐related risk factors. Although procedure‐related factors were major predictors of PEP, once it occurred, patient related factors (old age, male gender, and high comorbidity) were the major predictor of developing moderate–severe PEP.

Prior studies have shown that academic year‐end changeover has been implicated in increasing adverse events rate in several healthcare settings.^18^, ^19^, ^20^ It is reasonable to conclude that this effect will be prominent in the case of PEP risk, as many of the identified risk factors (such as difficult cannulation attempts and inadvertent instrumentation of pancreatic duct) are related to endoscopist skills and experience.^1^, ^8^, ^9^, ^21^ However, the reports that looked specifically at the endoscopist or center volume and the involvement of a trainee as risks factors for development of PEP have been inconsistent. One study reported significant increase in the odds ratio of PEP with trainee involvement.^22^ Other larger studies concluded that the risk of PEP was not affected by case volume (of either the single endoscopist or the center), by trainee involvement, or by the month of the procedure.^1^, ^10^, ^14^, ^23^

Our study shows that urban‐teaching hospitals had lower PEP rate compared with urban‐nonteaching along with no variation observed across the monthly rate of PEP suggesting the lack of the “July effect.” Our results also showed that among patients who were admitted to urban‐nonteaching hospitals, the PEP rate was slightly higher during July–December period (7%) compared with January–June (6.3%, *P* < 0.001). The presence of teaching faculty in the teaching hospitals might mitigate the theoretical risk that can result from low experience or trainee involvement. Additionally, better patient selection and adherence to guidelines in performing ERCP are more likely expected in an academic setting. This could potentially lower the overall rate of PEP in these hospitals.

Our analysis showed significant racial (Asians 10% and Hispanic 7.8%, compared with Caucasians 4.9%) and geographic (west 11.7%, compared with northeast 3.6%) differences in the rate of PEP. Prior meta‐analysis indicated regional variation in the rate of PEP across trials from North America, Europe, and Asia.^6^ The proportion of ERCPs across racial categories represents the distribution along the lines of racial breakdown of the United States population. However, none of the prior large, US‐based studies included sufficient racial or geographic diversity to be able to investigate this observation. National databases are ideal to explore these variations and have been used extensively in other conditions. These findings suggest the presence of additional demographic or genetic risk factors that are yet to be investigated.^24^

Female gender has been reported as risk factor for PEP with pooled OR of 1.4 (1.24–1.58) based on a recent meta‐analysis.^25^ However, several large studies have showed no increased risk.^1^, ^12^ Our analysis yielded almost equal PEP risk for females 5.5% compared with males 5.3%. Whereas younger age has been reported as risk factor for PEP on prior studies,^1^, ^9^, ^22^ other studies failed to demonstrate that association.^8^, ^12^, ^23^, ^25^ Our analysis showed minor differences across age groups that are probably not clinically relevant, although it achieved statistical significance due to large sample size.

Regarding the type of ERCP intervention, multivariate analysis showed that diagnostic cholangiogram, biliary stone removal, and biliary dilation carry no increase in the risk of PEP from the baseline. While sphincterotomy and biliary stent placement slightly increased the risk of PEP, all pancreatic intervention had higher risk of PEP. Sphincter of Oddi manometry had the highest risk of PEP (OR 2.5, *P* < 0.001) in the univariate and multivariate analysis. These results are identical or follow the same trend to previously published reports about PEP risk by the type of ERCP maneuver.^1^, ^12^, ^23^, ^25^

Furthermore, patient who underwent guide wire‐assisted cannulation of the PD (identified by the presence of a code of PD cannulation and lack of a code for pancreatography) had no increase in the rate of PEP (4.3%, OR 1.05, *P* = 0.9). As compared with PEP risk for those who had diagnostic pancreatography (6.7%, OR 1.6, *P* < 0.001). Our finding is almost identical to the reported reduction in the rate of PEP with using a guide wire‐assisted cannulation compared to contrast‐assisted cannulation that was reported in a prior meta‐analysis of randomized clinical trials (3.5% *vs* 6.7%, respectively).^26^

Pancreatic stent protective role disseminated in the late 1990s and early 2000.^3^ Thus, our data represent the era post wide use of pancreatic stent for patients with high risk. The observed higher PEP rate among patients who received pancreatic stent reflects selection of patients with high risk as the retrospective design is not suited to evaluate the efficacy of therapeutic interventions.

In our analysis, 17% of patients with PEP (0.9% out of all ERCPs) were classified as having moderate–severe due to the presence of major organ failure or sepsis. This rate is close to the previously reported rate of 24%.^12^ However, recent meta‐analysis of placebo or nonstent arms of randomized controlled trials showed the rate of moderate–severe PEP to be as high as 31%.^6^ Likely the differences in the reported rates are attributed to the differences in defining or identifying PEP. The same meta‐analysis showed similar risk of moderate–severe PEP among low and high‐risk groups (based on classic risk factors of young, female, SOD manometry).^6^ This is consistent to our results, which additionally showed that advanced age, male gender, and higher comorbidity were the major predictors of PEP severity.

The authors acknowledge possible limitations of this study. The study is based on administrative retrospective database analysis. There is lack of information about intravenous fluid hydration and the use of rectal indomethacin, which could affect the risk of PEP. But having large sample size will likely average the effect of these interventions across the population. Additionally, ICD 9 code does not differentiate between endoscopic biliary, pancreatic, or precut sphincterotomy or papillotomy. Thus, all these techniques were labeled as biliary sphincterotomy as it represents the most common intervention. There is no specific code for necrotizing pancreatitis in ICD 9 system, and thus these patients would be classified as mild PEP if they did not have major organ failure.

The strength of this study lies in the large number of patients included; to our knowledge, this is the largest study addressing PEP to date. Additionally, since this study was based on nationwide data rather than data from centers of excellence, the results are more representative for the general population. Furthermore, our study expands the current knowledge about the predictors of PEP and its severity and suggests novel risk factors. Due to the large number of variables that make each patient and ERCP unique, this study does not provide an exhaustive review of all factors that may lead to the development of PEP. This study does, however, present a review of several factors using a large national database.

## Conclusion

Our study examined the predictors of PEP using a large national database. The study confirmed the previously reported ERCP maneuvers that are associated with increasing PEP risk (SOD manometry, pancreatic contrast injection, and pancreatic interventions). Additionally, it showed that there was no increase in the risk of PEP early in the academic year in teaching hospitals. Furthermore, it identified racial and geographic variations of PEP rate that has not previously reported. And finally, the analysis showed that patient related factors (old age, male gender, and high comorbidity) were the major predictor of developing moderate–severe PEP.

## Declaration of conflict of interest

All authors have no conflicts of interest to disclose.

## Author Contribution

Ali Abbas involved in study concept and design; acquisition of data; analysis and interpretation of data; drafting of the manuscript; critical revision of the manuscript for important intellectual content; statistical analysis. Sajiv Sethi involved in study concept and design; acquisition of data; analysis and interpretation of data; drafting of the manuscript; critical revision of the manuscript for important intellectual content; statistical analysis. Gitanjali Vidyarthi involved in study concept and design; administrative and technical support; study supervision. Pushpak Taunk involved in study concept and design; administrative and technical support; study supervision.

## Supporting information


**Data S1.** Supporting information.

## References

[1] Testoni PA , Mariani A , Giussani A *et al* Risk factors for post‐ERCP pancreatitis in high‐ and low‐volume centers and among expert and non‐expert operators: a prospective multicenter study. Am. J. Gastroenterol. 2010; 105: 1753–61. 20372116 10.1038/ajg.2010.136

[2] Christensen M , Matzen P , Schulze S , Rosenberg J . Complications of ERCP: a prospective study. Gastrointest. Endosc. 2004; 60: 721–31. 15557948 10.1016/s0016-5107(04)02169-8

[3] Freeman ML , Guda NM . Prevention of post‐ERCP pancreatitis: a comprehensive review. Gastrointest. Endosc. 2004; 59: 845–64. 15173799 10.1016/s0016-5107(04)00353-0

[4] Anderson MA , Fisher L , Jain R *et al* Complications of ERCP. Gastrointest. Endosc. 2012; 75: 467–73. 22341094 10.1016/j.gie.2011.07.010

[5] Thaker AM , Mosko JD , Berzin TM . Post‐endoscopic retrograde cholangiopancreatography pancreatitis. Gastroenterol Rep (Oxf). 2015; 3: 32–40. 25406464 10.1093/gastro/gou083 PMC4324870

[6] Kochar B , Akshintala VS , Afghani E , et al. Incidence, severity, and mortality of post‐ERCP pancreatitis: a systematic review by using randomized, controlled trials. Gastrointest Endosc. 2015;81:143‐149.e149. 25088919 10.1016/j.gie.2014.06.045

[7] Elmunzer BJ , Scheiman JM , Lehman GA *et al* A randomized trial of rectal indomethacin to prevent post‐ERCP pancreatitis. N. Engl. J. Med. 2012; 366: 1414–22. 22494121 10.1056/NEJMoa1111103 PMC3339271

[8] Masci E , Mariani A , Curioni S , Testoni PA . Risk factors for pancreatitis following endoscopic retrograde cholangiopancreatography: a meta‐analysis. Endoscopy. 2003; 35: 830–4. 14551860 10.1055/s-2003-42614

[9] Wang P , Li ZS , Liu F *et al* Risk factors for ERCP‐related complications: a prospective multicenter study. Am. J. Gastroenterol. 2009; 104: 31–40. 19098846 10.1038/ajg.2008.5

[10] Freeman ML , Nelson DB , Sherman S *et al* Complications of endoscopic biliary sphincterotomy. N. Engl. J. Med. 1996; 335: 909–18. 8782497 10.1056/NEJM199609263351301

[11] Halttunen J , Meisner S , Aabakken L *et al* Difficult cannulation as defined by a prospective study of the Scandinavian Association for Digestive Endoscopy (SADE) in 907 ERCPs. Scand. J. Gastroenterol. 2014; 49: 752–8. 24628493 10.3109/00365521.2014.894120

[12] Cotton PB , Garrow DA , Gallagher J , Romagnuolo J . Risk factors for complications after ERCP: a multivariate analysis of 11,497 procedures over 12 years. Gastrointest. Endosc. 2009; 70: 80–8. 19286178 10.1016/j.gie.2008.10.039

[13] (HCUP) HCaUP. *Nationwide Inpatient Sample* Available from URL: http://www.hcup-us.ahrq.gov/nisoverview.jsp. Accessed 2/1/2018.

[14] Schulman AR , Abougergi MS , Thompson CC . Assessment of the July effect in post‐endoscopic retrograde cholangiopancreatography pancreatitis: Nationwide Inpatient Sample. World J Gastrointest Endosc. 2017; 9: 296–303. 28744341 10.4253/wjge.v9.i7.296 PMC5507820

[15] Inamdar S , Berzin TM , Sejpal DV *et al* Pregnancy is a risk factor for pancreatitis after endoscopic retrograde cholangiopancreatography in a National Cohort Study. Clin. Gastroenterol. Hepatol. 2016; 14: 107–14. 25952311 10.1016/j.cgh.2015.04.175

[16] Bradley EL . A clinically based classification system for acute pancreatitis. Summary of the International Symposium on Acute Pancreatitis, Atlanta, Ga, September 11 through 13, 1992. Arch. Surg. 1993; 128: 586–90. 8489394 10.1001/archsurg.1993.01420170122019

[17] Charlson ME , Pompei P , Ales KL , MacKenzie CR . A new method of classifying prognostic comorbidity in longitudinal studies: development and validation. J. Chronic Dis. 1987; 40: 373–83. 3558716 10.1016/0021-9681(87)90171-8

[18] Young JQ , Ranji SR , Wachter RM , Lee CM , Niehaus B , Auerbach AD . "July effect": impact of the academic year‐end changeover on patient outcomes: a systematic review. Ann. Intern. Med. 2011; 155: 309–15. 21747093 10.7326/0003-4819-155-5-201109060-00354

[19] Barzansky B , Etzel SI . Medical schools in the United States, 2007‐2008. JAMA. 2008; 300: 1221–7. 18780853 10.1001/jama.300.10.1221

[20] Haller G , Myles PS , Taffé P , Perneger TV , Wu CL . Rate of undesirable events at beginning of academic year: retrospective cohort study. BMJ. 2009; 339: b3974. 19826176 10.1136/bmj.b3974 PMC2762036

[21] Ding X , Zhang F , Wang Y . Risk factors for post‐ERCP pancreatitis: a systematic review and meta‐analysis. Surgeon. 2015; 13: 218–29. 25547802 10.1016/j.surge.2014.11.005

[22] Cheng CL , Sherman S , Watkins JL *et al* Risk factors for post‐ERCP pancreatitis: a prospective multicenter study. Am. J. Gastroenterol. 2006; 101: 139–47. 16405547 10.1111/j.1572-0241.2006.00380.x

[23] Freeman ML , DiSario JA , Nelson DB *et al* Risk factors for post‐ERCP pancreatitis: a prospective, multicenter study. Gastrointest. Endosc. 2001; 54: 425–34. 11577302 10.1067/mge.2001.117550

[24] Mounzer R , Whitcomb DC . Genetics of acute and chronic pancreatitis. Curr. Opin. Gastroenterol. 2013; 29: 544–51. 23872486 10.1097/MOG.0b013e3283639383 PMC5654556

[25] Chen JJ , Wang XM , Liu XQ *et al* Risk factors for post‐ERCP pancreatitis: a systematic review of clinical trials with a large sample size in the past 10 years. Eur. J. Med. Res. 2014; 19: 26. 24886445 10.1186/2047-783X-19-26 PMC4035895

[26] Tse F , Yuan Y , Moayyedi P , Leontiadis GI . Guide wire‐assisted cannulation for the prevention of post‐ERCP pancreatitis: a systematic review and meta‐analysis. Endoscopy. 2013; 45: 605–18. 23807804 10.1055/s-0032-1326640

