# Supplementary material for: Predictors of postendoscopic retrograde cholangiopancreatography pancreatitis, analysis of more than half a million procedures performed nationwide over the last 15 years
Source: JGH Open. 2020 Apr 17;4(4):736–42. doi: 10.1002/jgh3.12341 (PMC7411627; doi:10.1002/jgh3.12341)

Appendix for Revision

| **Table 1: ERCP intervention type by hospital location.** | | | | | | |
| --- | --- | --- | --- | --- | --- | --- |
|  | **Rural** | | **Urban nonteaching** | | **Urban teaching** | |
|  | **# ERCP** | **%** | **# ERCP** | **%** | **# ERCP** | **%** |
| **Diagnostic ERCP** | 6,756 | **15.2%** | 30,245 | **10.4%** | 33,020 | **10.4%** |
| **Biliary intervention** | 36,198 | 81.2% | 252,448 | 86.7% | 269,715 | 85.2% |
| **Pancreatic intervention** | 279 | 0.6% | 1,311 | 0.5% | 1,590 | 0.5% |
| **Both biliary and pancreatic** | 1,342 | 3.0% | 7,055 | 2.4% | 12,225 | 3.9% |


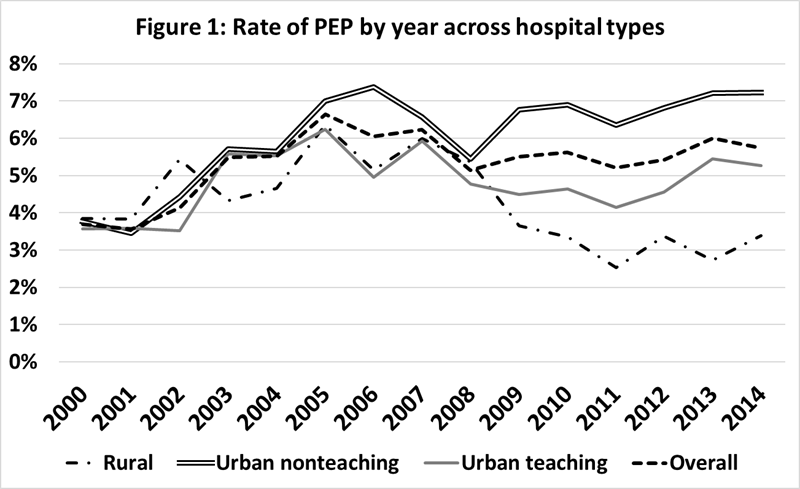


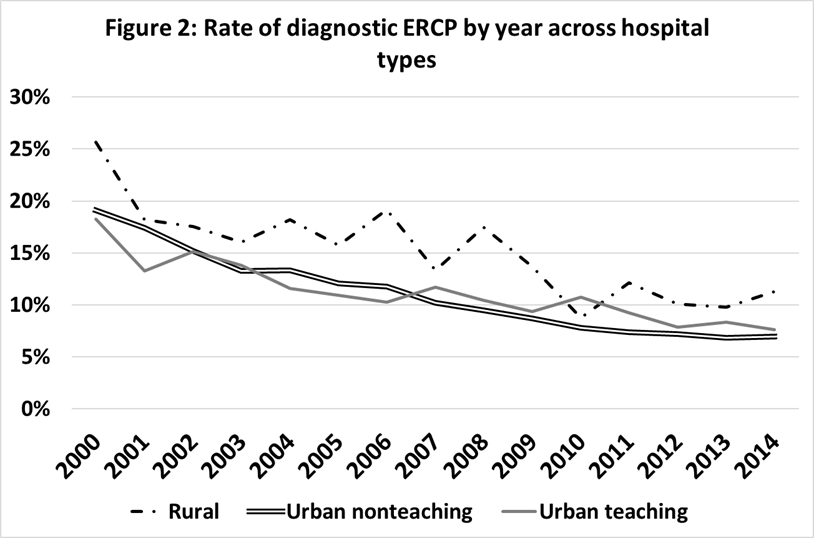

Supplement: Supplementary file 1 — Data S1. Supporting information. [file JGH3-4-736-s001.docx]
